# Incorporating Inflammation Biomarker‐Driven Multivariate Predictive Model for Coronary Microcirculatory Dysfunction in Acute Myocardial Infarction Following Emergency Percutaneous Coronary Intervention

**DOI:** 10.1002/clc.70032

**Published:** 2024-10-21

**Authors:** Zhuoya Yao, Bin Ding, Jun Wang, Shaohuan Qian, Xilong Song, Yao Li, Siyu Ding, Hongju Wang, Miaonan Li

**Affiliations:** ^1^ Department of Cardiovascular Disease The First Affiliated Hospital of Bengbu Medical University Bengbu China

**Keywords:** acute myocardial infarction, cardiac magnetic resonance, coronary microcirculatory dysfunction, inflammation marker, predictive model

## Abstract

**Background:**

Despite patients with successful revascularization as evidenced by angiographic findings, inadequate clinical management of coronary microcirculatory dysfunction (CMD) may result in preventable adverse outcomes. Therefore, it is imperative to use a multimodal data‑driven predictive model for the occurrence of CMD in patients with acute myocardial infarction (AMI) following emergency percutaneous coronary intervention (PCI).

**Methods:**

A prospective case−control analysis was conducted on a cohort of 77 patients with AMI who underwent PCI. The most informative predictors were selected for the predictive model through the application of LASSO analysis and multi‐factor logistic regression. The diagnosis of CMD is based on findings from cardiac magnetic resonance (CMR).

**Results:**

Based on the findings from LASSO analysis and multi‐factor logistic regression, variables including sex, neutrophil‐to‐lymphocyte ratio (NLR), Gensini score, and diabetes mellitus were identified as independent predictors for the development of CMD in AMI patients who underwent emergency PCI. The predictive model was evaluated using bootstrap self‐sampling 500 times. The resulting predictive model demonstrated an AUC value of 0.897 (95% CI: 0.827−0.958). The calibration curves exhibited good concordance between the predictions generated by the model and the CMR analysis. Furthermore, decision curve analysis revealed that the predictive model provided valuable clinical benefit in predicting CMD.

**Conclusions:**

The multivariate predictive model, constructed using readily available clinical variables in patients with AMI who underwent PCI, demonstrates satisfactory predictability for identifying comorbid CMD, thereby facilitating the identification of high‐risk patients.

## Graphical abstract

A prospective case−control analysis was conducted on a cohort of 77 patients with AMI who underwent PCI. The most informative predictors were selected for the predictive model through the application of LASSO analysis and multi‐factor logistic regression. The diagnosis of CMD is based on findings from cardiac magnetic resonance (CMR). Based on the findings from LASSO analysis and multi‐factor logistic regression, variables including sex, neutrophil‐to‐lymphocyte ratio (NLR), Gensini score, and diabetes mellitus were identified as independent predictors for the development of CMD in AMI patients who underwent emergency PCI. The multivariate predictive model, constructed using readily available clinical variables in patients with AMI who underwent PCI, demonstrates satisfactory predictability for identifying comorbid CMD, thereby facilitating the identification of high‐risk patients.

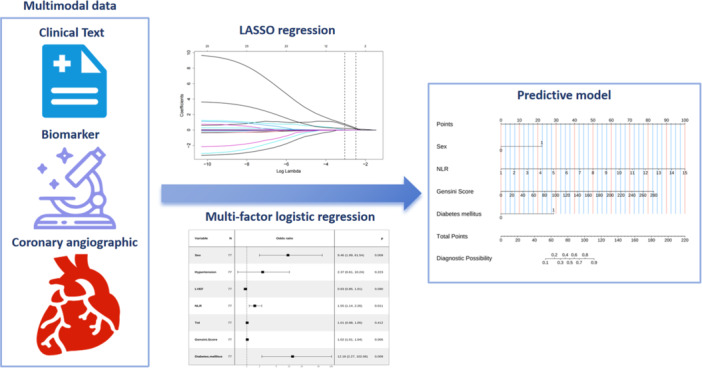

## 1 Introduction

1.1

Acute myocardial infarction (AMI), being a severe manifestation of coronary heart disease, poses a significant threat to individuals' lives and well‐being and stands as one of the primary causes contributing to cardiovascular mortality [1, 2, 3]. The current optimal treatment for AMI is blood flow reperfusion therapy, primarily performed through percutaneous coronary intervention (PCI) [1, 2, 3]. Notably, revascularization following AMI may not fully mitigate the presence of coronary microcirculatory dysfunction (CMD), as it persists in over 50% of AMI patients who undergo successful PCI [4, 5, 6]. Furthermore, CMD poses an elevated risk of major adverse cardiovascular events (MACEs) irrespective of the presence of epicardial coronary stenosis [7]. Consequently, the success of PCI is heavily reliant on the structural and functional integrity of the coronary microcirculation, particularly in patients with AMI following emergency PCI. Thus, the evaluation of CMD in patients with AMI undergoing post‐emergency PCI is a crucial objective in the management of AMI following PCI, and the timely diagnosis of CMD represents a potential focal point for clinical care.

The etiology of CMD is multifaceted and diverse, with overlapping etiologic factors complicating the differentiation of specific pathogenic mechanisms in clinical practice. Furthermore, a multitude of factors, including diabetes mellitus, hypertension, and excessive inflammatory response, exert influence on coronary microcirculatory function, thereby further complicating the comprehensive clinical assessment of CMD [8, 9]. Currently, the lack of systematic and standardized detection methods for CMD poses significant challenges to the systematic prevention and comprehensive management of AMI. Therefore, enhancing the detection methods for CMD can facilitate the provision of personalized treatment plans for patients. Cardiac magnetic resonance imaging (CMR) is presently the predominant and precise method utilized for the measurement and assessment of CMD in clinical settings [10, 11, 12]. As a noninvasive and radiation‐free assessment tool, this method provides a comprehensive evaluation of coronary vascular anatomy, function, perfusion, and tissue characteristics. Moreover, it facilitates the identification of microvascular lesions, which is pivotal for informing clinical treatment decisions and predicting outcomes in post‐PCI patients. However, there exists a significant disparity in the allocation of medical resources within China's primary healthcare institutions, characterized by inadequate availability of advanced medical equipment and technology in certain facilities, consequently leading to suboptimal levels of diagnosis and treatment. Despite the accurate assessment capabilities and crucial predictive value offered by machines like CMR for evaluating interventional procedures, not all primary hospitals are equipped with such sophisticated apparatus. Therefore, it is imperative to develop user‐friendly diagnostic and predictive tools that can be readily employed at a grassroots level in China, facilitating timely evaluation of CMD subsequent to PCI.

The inflammatory response, serving as the initiating and pivotal factor in the pathogenesis of coronary heart disease, exerts a significant influence on the formation of coronary atheromatous plaques and obstruction of myocardial microvasculature [13, 14, 15]. Recently, it has been demonstrated that elevated inflammation‐related markers such as leukocytes and neutrophils during AMI are associated with larger infarct size and extensive CMD [16, 17]. Therefore, this study aims to integrate the findings from combining inflammatory biomarkers with clinically accessible multimodal data to establish a scoring model for CMD occurrence in patients with AMI after emergency PCI. This model will serve as a novel basis for assessing microcirculation function and facilitating early disease intervention.

## 2 Methods

### 2.1 Study Design and Participants

We prospectively enrolled consecutive patients with AMI who underwent successful emergency PCI in our department from June 2022 to December 2023. All patients underwent successful CMR imaging 5−7 days after PCI to assess the presence of CMD. The diagnostic criteria for AMI were based on the latest recommendations of the Acute Coronary Syndromes Guideline [3]. Patients were excluded if they had any of the following clinical conditions: previous history of cardiac surgery, along with frequent pre‐systole, sustained ventricular tachycardia, or other tachyarrhythmias; prior myocardial infarction, cardiac valvular disease; significant liver and renal function abnormalities; malignant tumors; infectious diseases within the past month; pregnancy status; mental anomalies, poor cooperation, and difficulty in completing breath‐holding after a 0.5 h training session; allergy to gadolinium agents; presence of ferromagnetic objects; claustrophobia; and so forth.

The study protocol underwent review and approval by the Ethics Committee of Bengbu Medical University (No. [2023] KY046), ensuring compliance with ethical standards. Participants provided their consent through the completion of an informed consent form.

### 2.2 Laboratory Tests

Approximately 5 mL of fasting peripheral venous blood was aseptically collected from subjects before emergency coronary intervention using sodium heparin anticoagulated tubes and subsequently sent to a designated testing facility for analysis of serum inflammatory markers and other biochemical indices. The levels of serum inflammatory markers were determined as follows: neutrophil‐to‐lymphocyte ratio (NLR) = neutrophils/lymphocytes count; platelet‐to‐lymphocyte ratio (PLR) = platelets/lymphocytes count; systemic Inflammation Index (SII) = platelets × neutrophils/lymphocytes count [18, 19, 20].

### 2.3 Coronary Angiography and PCI

The procedure of coronary angiography was conducted by a cardiovascular medicine specialist at our institution, with the results evaluated based on the 2001 ACC/AHA Report on the Diagnosis and Treatment of Cardiovascular Disease [21]. Patients with confirmed coronary artery stenosis underwent coronary stenting, and detailed records were maintained of both the angiography and stenting procedures. The extent of stenosis was determined using the Gensini score in conjunction with the patient's angiographic results [22], with two experienced cardiologists independently assessing the severity.

### 2.4 CMR Examination Methods and Image Processing

**Figure 1:**
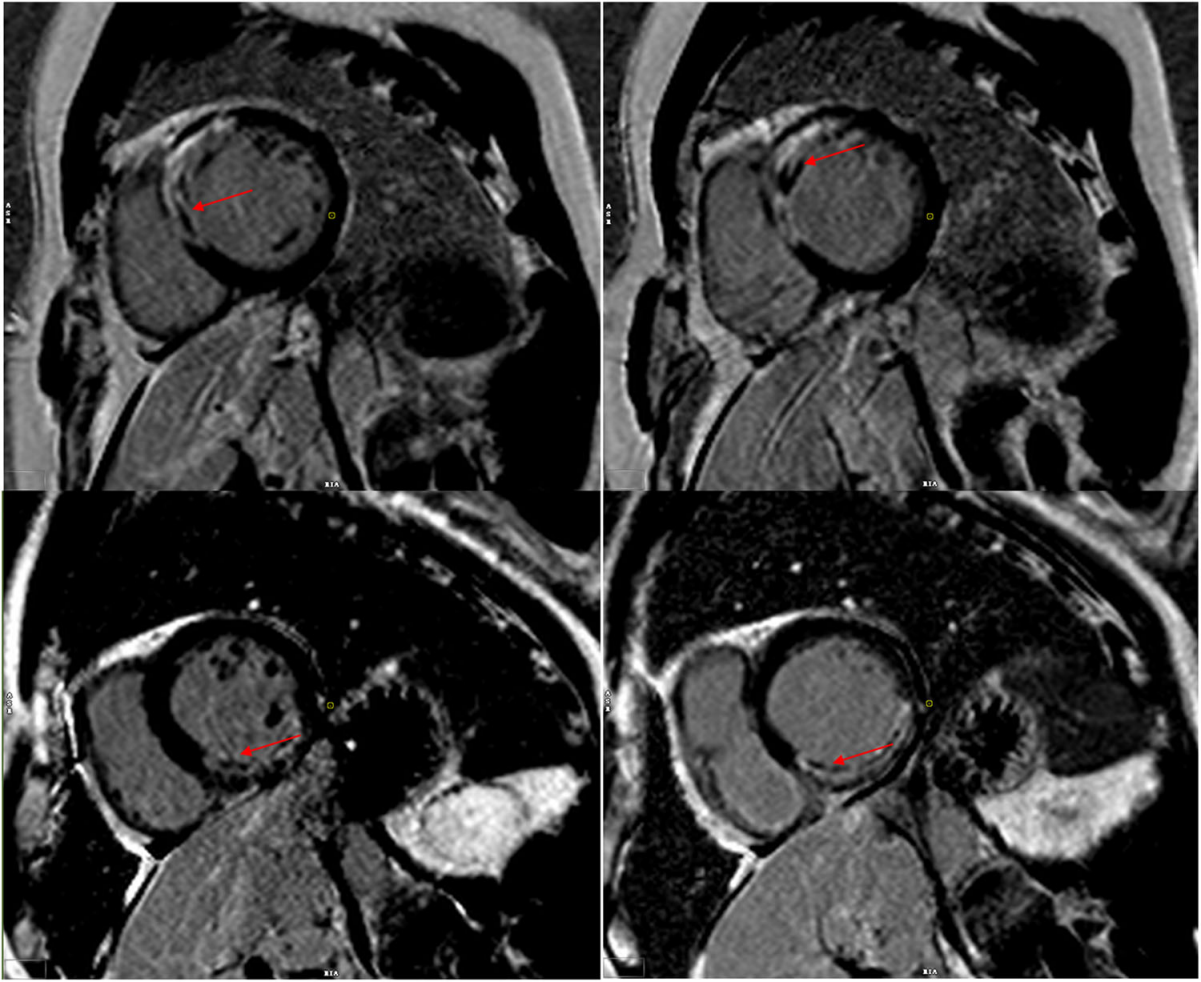
The cardiac magnetic resonance imaging reveals regions of diminished signal intensity in the enhancement, indicative of coronary microcirculatory dysfunction.

All patients underwent CMR imaging 5−7 days postoperatively, with images acquired at end‐expiration to capture steady‐state free precession cine sequences of each cardiac section. Gadolinium contrast delayed enhancement scanning was performed using a high‐pressure syringe to administer gadopentetate dextran injection (469 mg/mL × 20 mL) via a peripheral vein at a dose of 0.2 mmol/kg at a rate of 4.0 mL/s, followed by an equivalent volume of 0.9% sodium chloride injection at the same rate. The cardiac gated breath‐hold phase‐sensitive inversion recovery sequence scanning was initiated 10 min after contrast administration. All images were transferred into CVI42 software by one cardiologist for cardiac function as well as postprocessing by CMR tissue tracking technique to assess CMD. CMD definition labeled: delayed enhancement of nonenhancing regions within the myocardium [23] (Figure 1).

### 2.5 Statistical Analysis

The data were analyzed and processed using SPSS 23 and R software 4.2.2. Measurement data were presented as mean ± standard deviation, with normally distributed data subjected to ANOVA analysis and non‐normally distributed data tested using nonparametric tests. Categorical data were described using constituent ratios, and comparisons were conducted using the *χ* ^2^ test. The risk factors for microcirculatory disorders following PCI in patients with ST‐segment elevation myocardial infarction (STEMI) were analyzed using LASSO and multifactorial logistic regression. Subsequently, a nomogram model was constructed by integrating the “rms” software package in R version 4.2.2. To validate the accuracy of our prediction model, we performed bootstrap resampling on a sample size of 500 individuals. Receiver operating characteristic (ROC) curves were employed to assess the discriminatory ability of the model, while the area under the curve (AUC) was calculated as a measure of its performance [24]. Furthermore, calibration curves were utilized to evaluate how closely the predicted occurrence of CMD aligned with CMR analysis [24]. Additionally, decision curve analysis (DCA) was conducted to evaluate the clinical efficacy of our model [24]. A significance level of *p* < 0.05 was considered for all statistical comparisons.

## 3 Results

### 3.1 Clinical Features of the Study Population

Of the 77 patients with AMI who underwent successful emergency PCI and subsequent CMR imaging post‐PCI. Fifty‐four (70.1%) were male and 23 (29.9%) were female, with a mean age of 60.2 ± 12.4 years. Baseline data exhibited diabetes mellitus, cardiac troponin I, Gensini score, NLR, PLR, and SII in the CMD group compared to the levels in the no‐CMD group (all *p *< 0.05, Table 1).

**Table 1:** Comparison of baseline data between patients in the CMD group and those in the no‐CMD group.

Characteristic	All cohort	CMD	No‐CMD	*p*
		*N* = 43	*N* = 34	
Male sex, *n* (%)	54 (70.1%)	34 (79%)	20 (59%)	0.054
Age, years	60 ± 12	61 ± 12	60 ± 13	0.776
Hypertension, *n* (%)	43 (55.8%)	26 (60%)	17 (50%)	0.358
Diabetes mellitus, *n* (%)	23 (29.9%)	17 (40%)	6 (18%)	0.037
Current smoking, *n* (%)	48 (62.3%)	27 (63%)	21 (62%)	0.926
Body mass index, kg/m^2^	25.4 (22.5, 27.6)	25.4 (22.9, 27.4)	25.6 (22.0, 27.5)	0.886
Clinic time, h	5 (3, 10)	5 (4, 10)	4 (3, 10)	0.455
Fasting blood glucose, mmol/L	7.1 (5.6, 9.4)	7.6 (6.2, 9.5)	6.2 (5.5, 9.2)	0.181
Uric acid, μmol/L	328 (258, 392)	292 (253, 390)	351 (259, 400)	0.569
Creatinine, μmol/L	65 (54, 79)	64 (54, 82)	66 (55, 72)	0.627
Total cholesterol, mmol/L	4.58 ± 1.50	4.54 ± 1.48	4.62 ± 1.55	0.824
Triglyceride, mmol/L	1.49 (1.01, 2.52)	1.42 (0.93, 2.42)	1.54 (1.14, 2.54)	0.344
High‐density lipoprotein cholesterol, mmol/L	1.01 (0.88, 1.20)	0.97 (0.88, 1.17)	1.05 (0.88, 1.31)	0.183
Low‐density lipoprotein cholesterol, mmol/L	2.77 (2.13, 3.34)	2.73 (2.18, 3.32)	2.89 (2.00, 3.35)	0.941
Lipoprotein (a), mg/L	218 (100, 411)	222 (126, 407)	204 (59, 422)	0.400
Cardiac troponin I, ng/mL	11 (4, 28)	15 (8, 39)	5 (1, 14)	0.013
Gensini score	108 ± 57	132 ± 56	79 ± 42	< 0.001
Culprit vessel TIMI grade				0.162
Low‐risk		15 (35%)	19 (56%)	
Mid‐risk		19 (44%)	9 (26%)	
High‐risk		9 (21%)	6 (18%)	
Culprit vessel				0.026
Left anterior descending coronary artery, *n* (%)		24 (56%)	13 (38%)	
Left circumflex coronary artery, *n* (%)		13 (30%)	1 (3%)	
Right coronary artery, *n* (%)		13 (30%)	20 (59%)	
Neutrophil‐to‐lymphocyte ratio	5.5 (3.4, 8.4)	6.3 (4.7, 9.8)	3.8 (2.3, 5.9)	< 0.001
Platelet‐to‐lymphocyte ratio	137 (98, 181)	158 (112, 195)	113 (75, 144)	0.007
Systemic inflammation index	1062 (657, 1570)	1328 (844, 1919)	794 (477, 1359)	0.002

### 3.2 Prediction Nomogram

**Figure 2:**
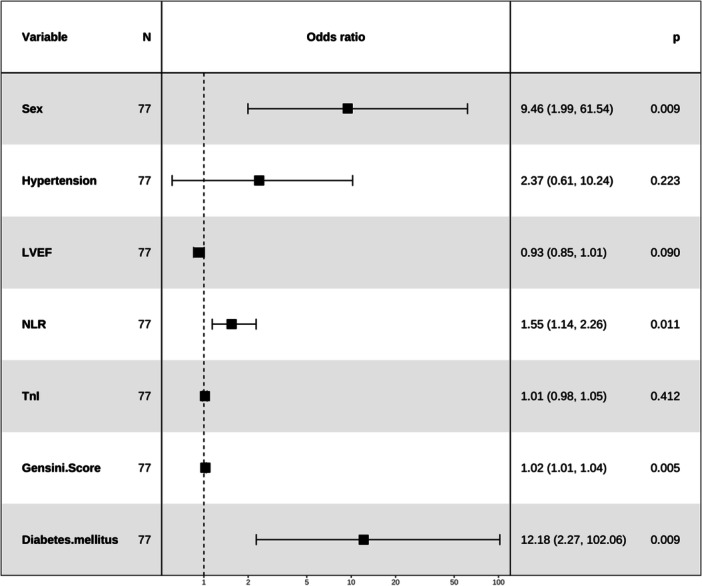
Forest plot with odd ratios, 95% confidence intervals, and corrected *p*‐values for independent variables identified by multivariate logistic regression analysis.

**Figure 3:**
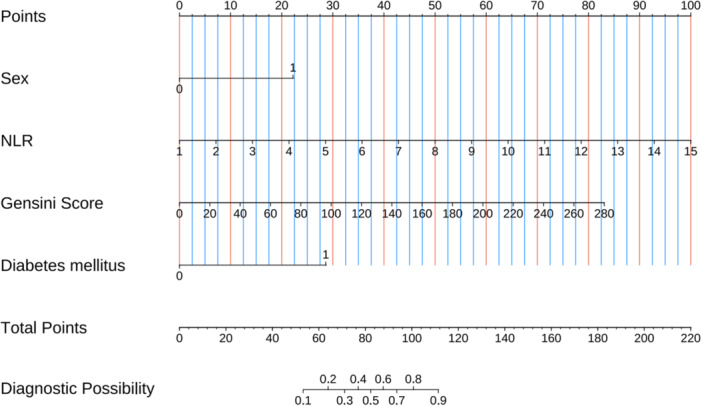
The predictive nomogram was constructed based on the optimal multivariate logistic regression model to estimate the probabilities of CMD.

The LASSO regression technique was employed to reduce the dimensionality of all variables (Table 1). To determine the optimal Lambda parameters, a 10‐fold cross‐validation approach was utilized. The Lambda value associated with the minimum cross‐validation error was considered as the optimal value for model selection. Subsequently, we determined the count of variables corresponding to non‐zero regression coefficients at this stage. The results of LASSO regression revealed that seven variables, namely sex, hypertension, left ventricular ejection fraction, NLR, cardiac troponin I, Gensini score, and diabetes mellitus, could significantly impact the occurrence of CMD after PCI for AMI (Supporting Information S1: Figures 1–2). The predictive strength of each variable is illustrated in the Supporting Information S1: Figure 3, which displays the correlation coefficients. Subsequently, a multivariate logistic regression analysis was conducted to identify predictive variables based on those presented in Figure 2 and establish the predictive model. A nomogram was constructed using four clinical routine variables, including sex, NLR, Gensini score, and diabetes mellitus (Figure 3).

### 3.3 Performance of the Predictive Nomogram

**Figure 4:**
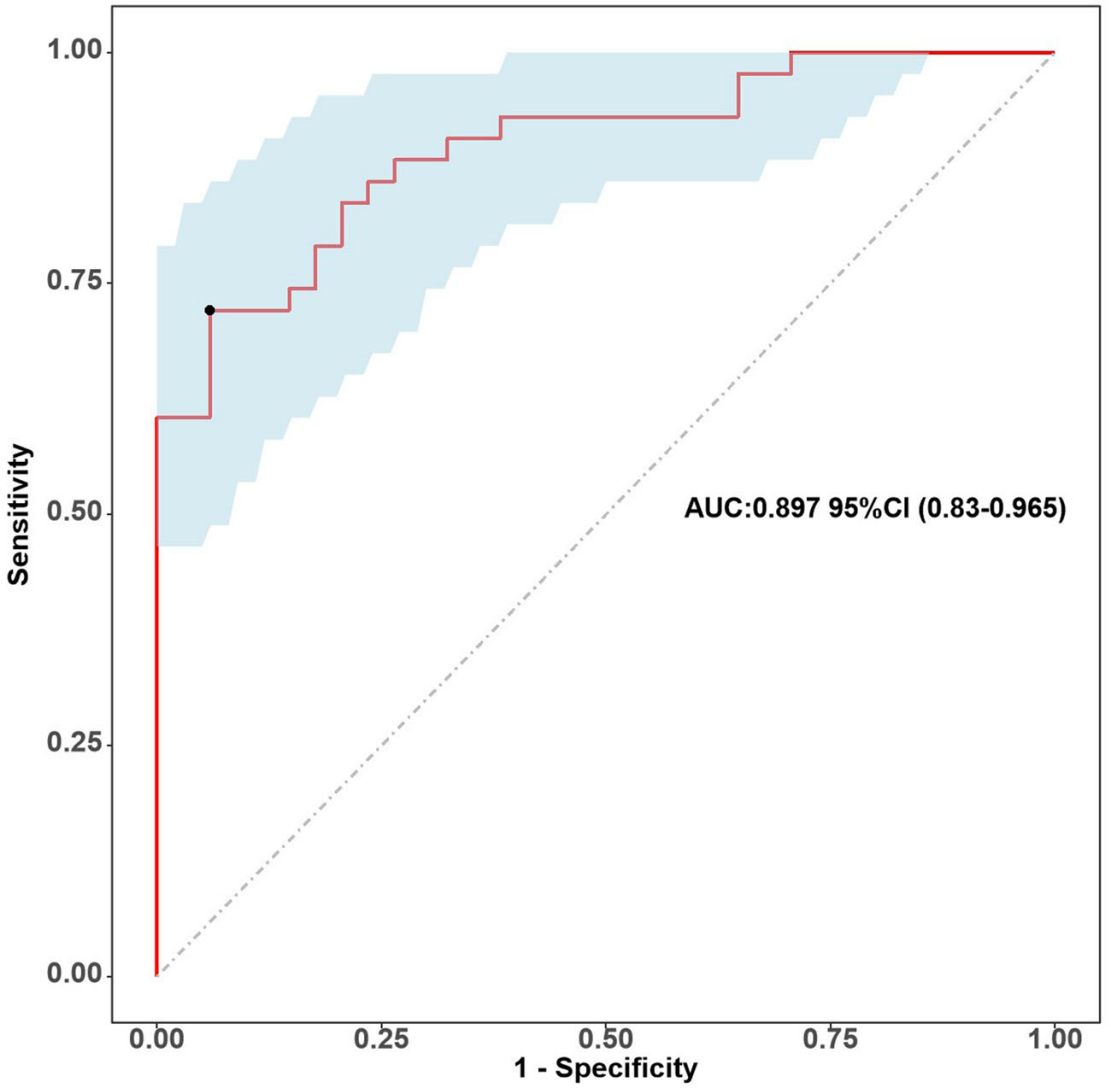
Receiver operating characteristic (ROC) curve analysis was performed with 500 bootstrap replications to validate the predictive efficacy of the nomogram in predicting CMD.

The utilization of bootstrap self‐sampling 500 times for internal validation represents a robust approach to comprehending the variability and confidence associated with model predictions. By employing this method, we conducted ROC analysis with 500 bootstrap replications, which revealed that the predictive nomogram exhibited superior discriminatory capability for CMD following PCI for AMI. This is evidenced by an impressive AUC value of 0.897, accompanied by a confidence interval ranging from 0.827 to 0.958 (Figure 4; Supporting Information S1: Figure 4). The calibration plots were constructed with 500 bootstrap replications to evaluate the agreement between the predicted risk of CMD in post‐PCI patients with AMI, as determined by the nomogram, and those confirmed by CMR analysis. (Supporting Information S1: Figures 5). The results of the predictive nomogram for the DCA with 500 bootstrap sampling are presented in Supporting Information S1: Figures 6, illustrating a substantial positive yield exhibited by this predictive nomogram. Additionally, the predictive nomogram demonstrated a higher net benefit rate than the individual models for the four independent variables (sex, NLR, Gensini score, and diabetes mellitus) in both the development and validation cohorts (Supporting Information S1: Figures 7).

## 4 Discussion

In our study, we employed machine learning to identify clinically accessible variables for predicting CMD following emergency PCI in patients with AMI. Subsequently, the performance of the predictive model was measured based on the concordance index (C‐index), the AUC, the calibration curve, and DCA using a bootstrap method with 500 resamples. This comprehensive model, encompassing clinical narratives, biomarker profiles, and imaging data, contributes to the development of a decision‐support tool for healthcare professionals. By accurately identifying high‐risk patients for CMD, clinicians can implement more aggressive anti‐inflammatory treatments, intensify monitoring efforts, or explore alternative therapeutic strategies. Importantly, the model's seamless integration into primary healthcare facilities promotes precise and personalized management of AMI patients who have undergone successful PCI, thereby potentially enhancing clinical outcomes.

AMI is a frequently encountered and reoccurring condition in clinical settings, serving as a severe manifestation of coronary heart disease distinguished by its sudden onset, brief duration, and elevated fatality rate, thereby exerting a profound impact on individuals' well‐being, health, and overall quality of life [25, 26, 27]. The complications arising from coronary microvascular dysfunction following an AMI, including infarct expansion, decline in cardiac contractility, and adverse cardiac remodeling, significantly impact the magnitude of myocardial injury, patient longevity, and quality of life [28, 29]. Previous studies have demonstrated that the prevalence of CMD in individuals with STEMI ranges from 50% to 60% [4, 5, 6]. CMD has been established as a reliable prognostic indicator for both short‐ and long‐term outcomes in patients with STEMI, accurately predicting adverse cardiac events such as cardiac death, recurrent myocardial infarction, and post‐infarction heart failure [30, 31, 32, 33, 34, 35]. Currently, it has been proposed that CMR may possess greater prognostic value than infarct size and ejection fraction in predicting adverse cardiovascular events [29, 36, 37]. Accumulated experimental findings support the unfavorable prognosis and increased prevalence of adverse cardiovascular events in individuals with cardiac diseases coexisting with CMD [29, 30, 31, 32, 33, 34, 35, 36, 37]. However, the clinical attention toward CMD is limited due to its reliance on diagnostic techniques such as CMR imaging. This poses difficulties for some primary healthcare settings that do not have access to this technology and for patients who may have an intolerance to MRI after undergoing PCI following a myocardial infarction.

Recently, there has been an increasing emphasis on the association between inflammation‐related mechanisms and CMD [38, 39]. The activation of inflammatory pathways subsequent to myocardial ischemia and necrosis resulting from occlusion of the coronary artery plays a pivotal role in the pathogenesis of CMD post‐myocardial infarction, as it facilitates tissue healing, scar formation, and ventricular remodeling [38]. Following the acute phase of myocardial infarction, the inflammatory response is primarily initiated by neutrophil infiltration and activation, leading to capillary and small artery compression within the coronary microcirculation due to interstitial myocardial edema. This process ultimately results in small artery spasm, microthrombosis, and subsequent development of CMD [39]. The presented study observed significantly elevated levels of serum inflammation markers, including NLR, PLR, and SII, in the group with CMD compared to the group without CMD at baseline. These findings suggest a potential role of serum inflammatory markers in both the progression and regression of CMD following PCI for AMI. Furthermore, employing machine learning techniques to select feature variables, followed by utilizing prediction nomogram for visualizing the degree of association between each variable and cardiovascular diseases [40, 41, 42, 43]. In our study, the incorporation of a multivariate predictive model driven by inflammation biomarkers could further optimize the management of patients with AMI undergoing emergency PCI. By integrating machine learning with clinical text, inflammation biomarkers, and imaging data, our predictive model provides an easy and reliable tool for identifying high‐risk patients who may develop CMD after PCI. The accurate prediction of CMD empowers clinicians to tailor therapeutic strategies with precision, potentially leading to enhanced patient outcomes in primary healthcare settings. For example, patients identified as being at higher risk for CMD may benefit from more aggressive anti‐inflammatory treatment, closer monitoring, or alternative therapeutic strategies that might not be considered in standard care protocols. The applicability of the present model in primary healthcare settings addresses a crucial gap in resource‐constrained environments where advanced imaging technologies such as CMR are not readily accessible. By relying on easily obtainable clinical and laboratory data, the model ensures that a wider range of healthcare facilities can provide personalized and evidence‐based care to patients with AMI, thereby democratizing access to advanced predictive tools in cardiovascular care. Consequently, the clinical implications of our predictive model are profound as it offers a pathway toward more individualized, precise, and accessible care for AMI patients at risk of CMD, ultimately aiming to alleviate the burden of cardiovascular morbidity and mortality.

## 5 Limitations

Initially, this portion of the research comprised a retrospective observational clinical study conducted at our institution, employing a relatively small sample size. Therefore, further validation is imperative to ascertain the accuracy and clinical relevance of the predictive model through a larger, multicenter sample. Moreover, our study does not directly provide prognostic information for AMI with different risks of post‐discharge clinical outcomes, the identification of CMD through our model can indirectly inform prognosis. Additionally, it is noteworthy that contemporary medical decision‐making heavily relies on predictive models; however, these models remain static over time despite the dynamic evolution observed in clinical regression for AMI due to advancements in treatment modalities, early detection techniques, and alterations in disease progression patterns. Consequently, as these factors continue to evolve, there is a possibility that the accuracy of predictive models may diminish. Finally, although internal validation methods were employed to assess the efficacy of the predictive model in this phase of the study, external validation should be considered as the gold standard for validating data set efficacy.

## 6 Conclusion

The present study established a diagnostic scoring model for CMD in patients with AMI undergoing PCI. This comprehensive model integrates easily accessible multimodal data obtained from the clinical setting, enabling the identification of high‐risk CMD patients. Such information can effectively guide adjustments to post‐PCI clinical treatment plans, ultimately leading to improved patient survival and enhanced quality of life. Consequently, this model holds great potential for optimizing post‐PCI clinical management and enhancing patient outcomes.

## Author Contributions

Shaohuan Qian, Xilong Song, Yao Li, and Siyu Ding conceived and performed the study. Jun Wang, Miaonan Li, and Jun Wang participated in the design of the study and performed the clinical study. Zhuoya Yao and Bin Ding wrote the manuscript and analysis and interpretation of the data. All authors agree to be accountable for all aspects of the work.

## Ethics Statement

This study was approved by the Ethics Committee of the First Affiliated Hospital of Bengbu Medical University ([2023]KY046).

## Consent

Written informed consent was obtained from all subjects (patients) in this study.

## Conflicts of Interest

The authors declare no conflicts of interest.

## Supporting information

Supporting information.

## Data Availability

Individual participant data that underlie the results reported in this article, after de‐identification, can be obtained from the corresponding author upon reasonable request.
