# Supplementary material for: Incorporating Inflammation Biomarker‐Driven Multivariate Predictive Model for Coronary Microcirculatory Dysfunction in Acute Myocardial Infarction Following Emergency Percutaneous Coronary Intervention
Source: Clin Cardiol. 2024 Oct 21;47(10):e70032. doi: 10.1002/clc.70032 (PMC11491760; doi:10.1002/clc.70032)

**Supplemental Figure 1**. Least absolute shrinkage and selection operator (LASSO) regression plot illustrating the trendlines of model coefficients for clinical variables potentially associated with the occurrence of coronary microvascular dysfunction (CMD) in patients with acute myocardial infarction (AMI) who underwent successful emergency percutaneous coronary intervention (PCI).


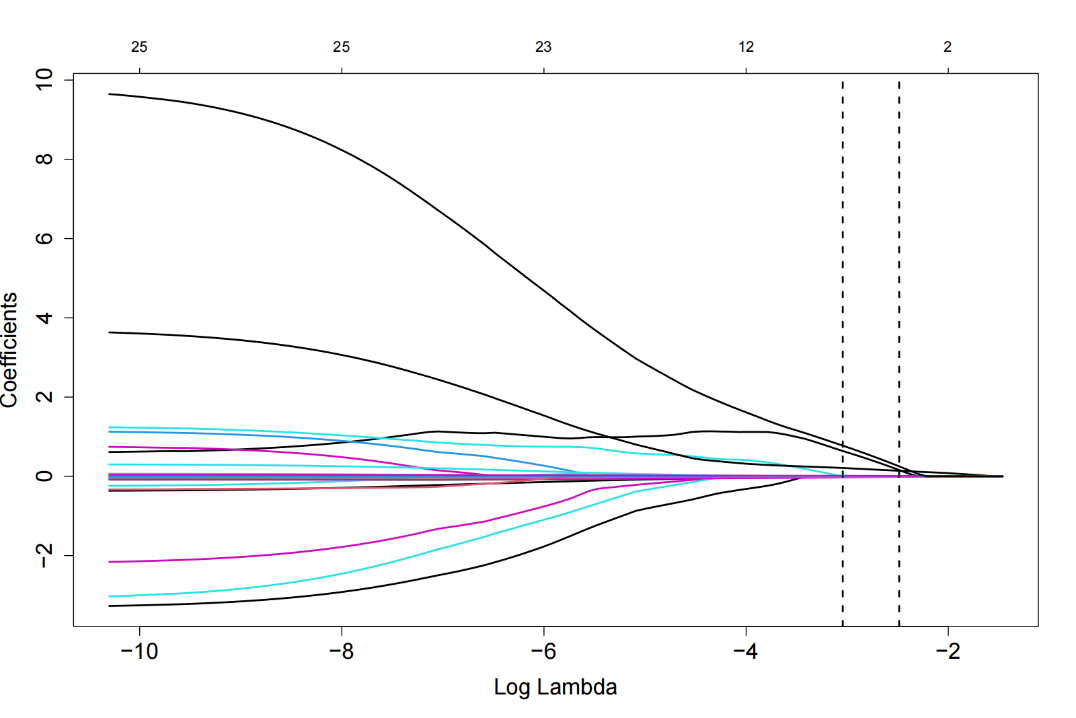


**Supplemental Figure 2**. The tuning parameter (λ) selection was performed using a cross-validation error curve with 10-fold cross-validation. Optimal values were identified based on the criteria of minimum and one standard error, as indicated by vertical lines.


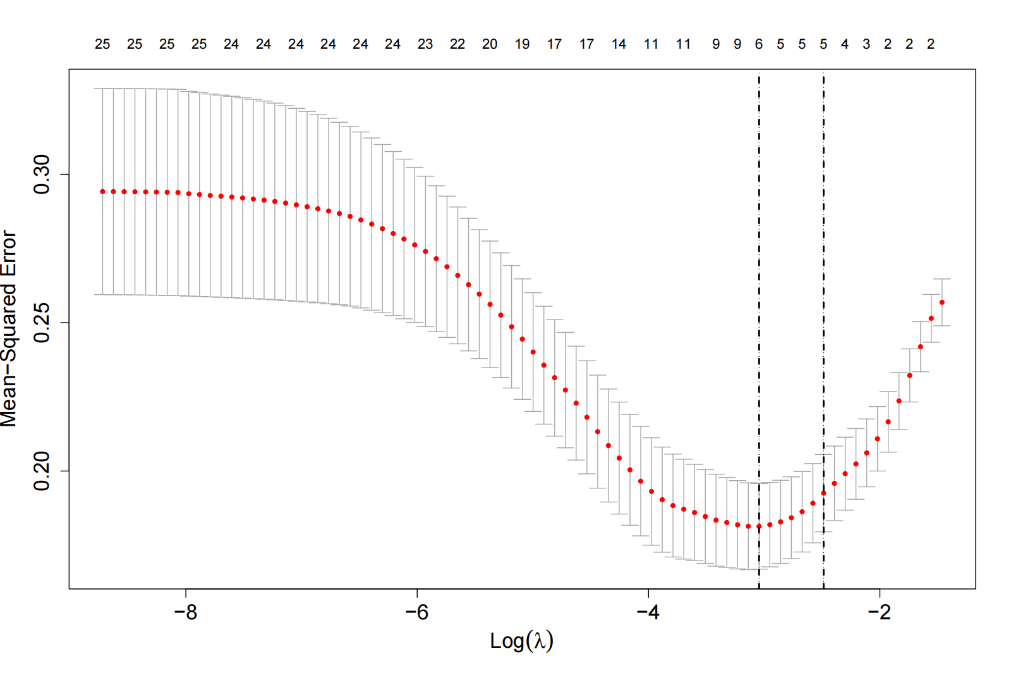


**Supplemental Figure 3**. Strength of correlation between variable and the occurrence of CMD according to correlation coefficients based on varying values of λ one standard error.


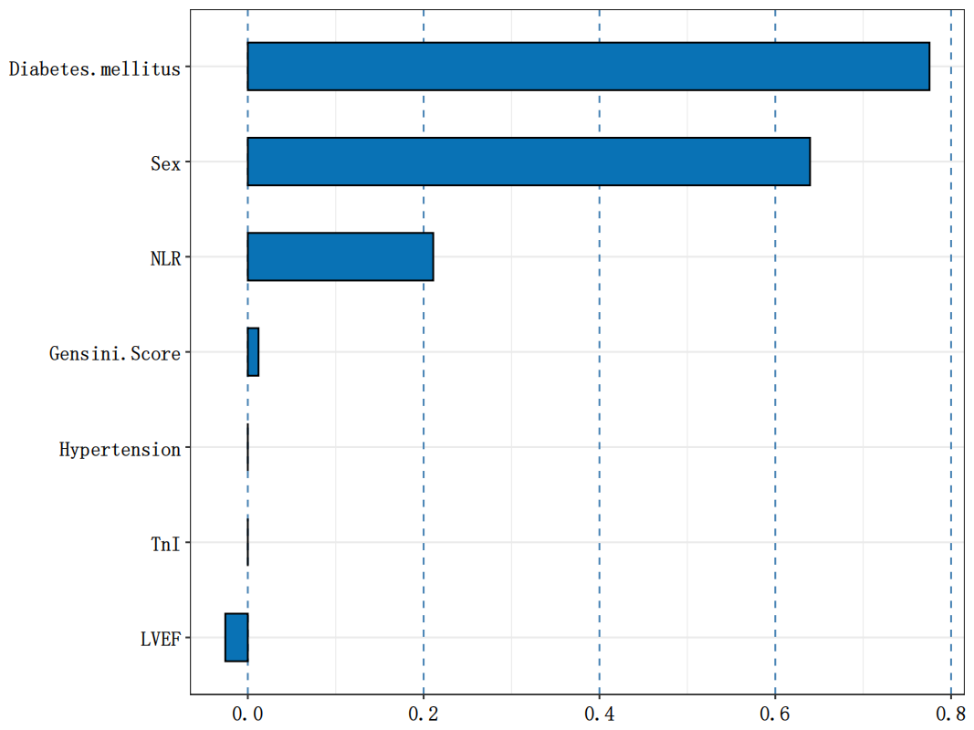


**Supplemental Figure 4**. ROC curve analysis was performed to evaluate the predictive nomogram and individual models for the four independent variables (sex, NLR, Gensini Score and diabetes mellitus) .


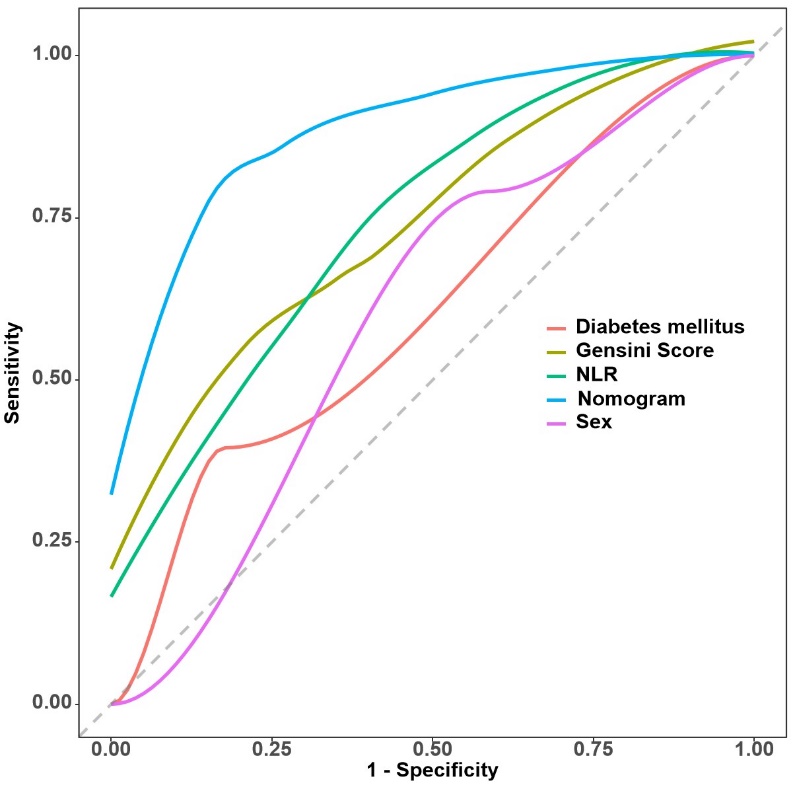


**Supplemental Figure 5**. Calibration curves with 500 bootstrap replications for the predictive efficacy of the predictive nomogram .


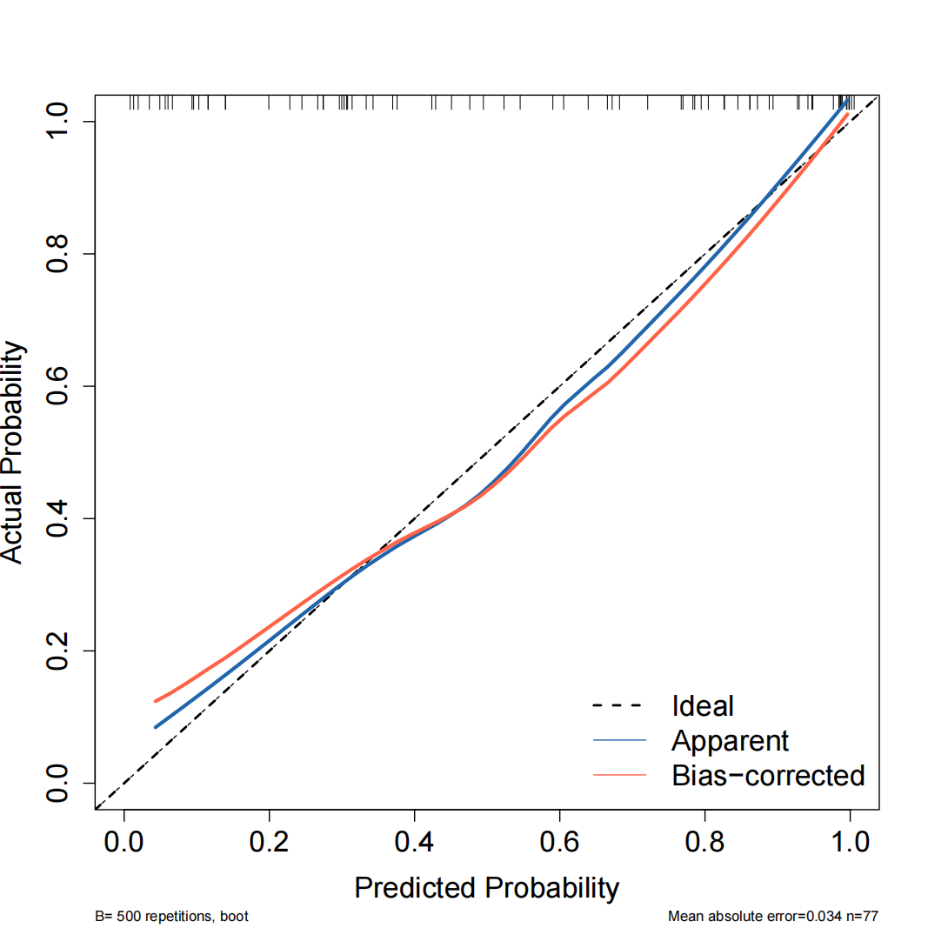


**Supplemental Figure 6**. Decision curve analysis of the predictive nomogram’s ability to predict the risk of CMD.


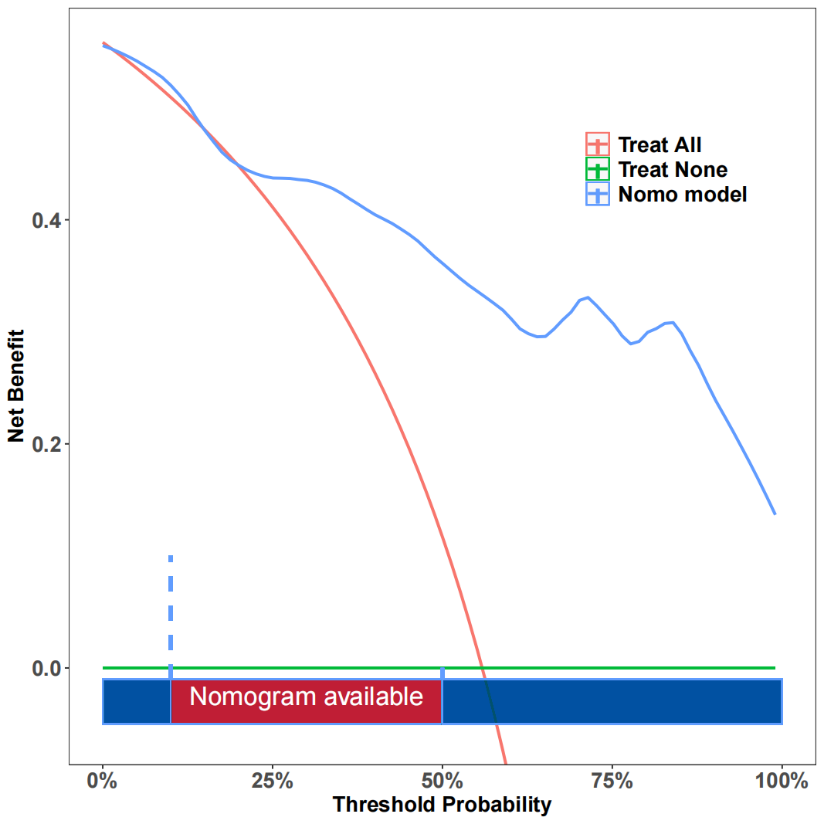


**Supplemental Figure 7**. Net benefit rate of the prediction nomogram and separate models for the four independent variables (sex, NLR, Gensini Score and diabetes mellitus) .


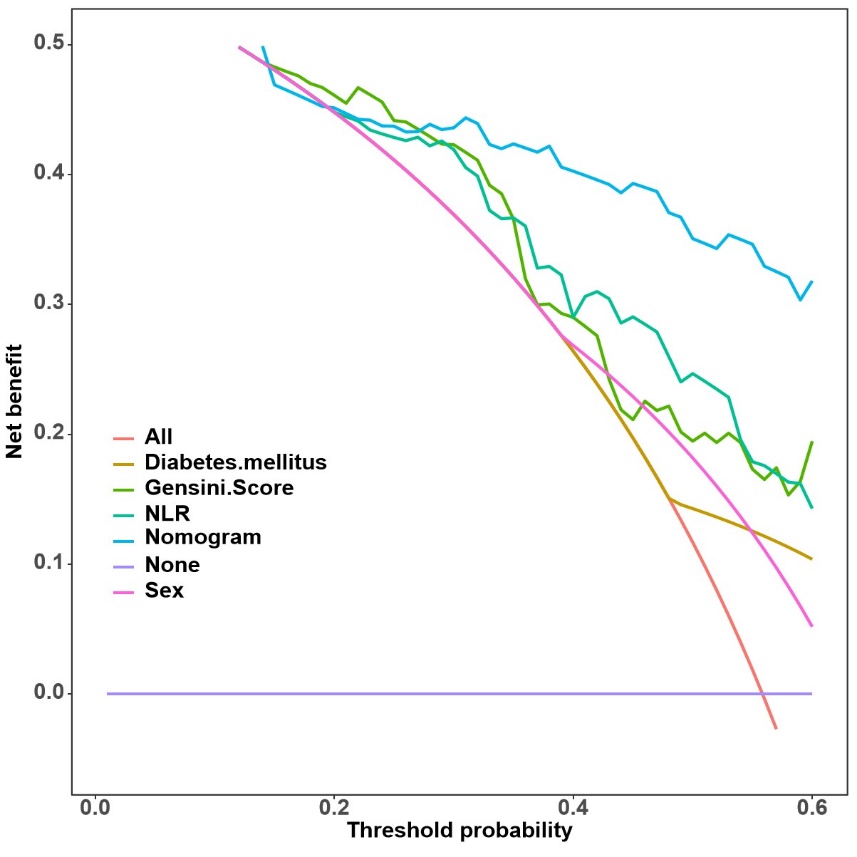

Supplement: Supplementary file 1 — Supporting information. [file CLC-47-e70032-s001.docx]
